# Awareness of undergraduate dental students, interns, and freshly graduated dentists about oral manifestation associated with COVID-19

**DOI:** 10.1186/s12903-023-03666-9

**Published:** 2023-12-09

**Authors:** Suliman Alrajhi, Maram Shalabi, Abdulaziz Alrajhi, Tamim Almarshud, Mohammed Almuhaysin, Abdullah Alhudaithi, Ahmed Fahad Alharbi, Nasser Alamri, Abdulaziz Alhumaid, Ali Aljuailan, Sultan Algefari, Suzan Salem, Islam Saad

**Affiliations:** 1 College of Dentistry, Qassim University, Buraydah, Saudi Arabia; 2 Department of Biochemistry, Faculty of Veterinary Medicine, Zagazig University, Sharqia Governorate, 4511 Egypt; 3 Department of Oral and Maxillofacial Surgery, Faculty of Dentistry, Alexandria University, Alexandria Governorate, Egypt; 4 Department of Oral Medicine, Oral Diagnosis and Radiology, Faculty of Dentistry, Alexandria University, Periodontology, Egypt

**Keywords:** Awareness, Dental students, Dentists, Oral manifestation, COVID-19, Saudi Arabia

## Abstract

**Objective:**

There is growing evidence supporting the presence of oral manifestations associated with COVID-19. The study investigates the knowledge of dental undergraduates and recently graduated dentists concerning oral presentations related to COVID-19.

**Methods:**

A cross-sectional descriptive study in Saudi Arabia comprised 305 individuals, including undergraduate dental students, interns, and freshly graduated dentists. Data were collected using a questionnaire disseminated to approximately 500 subjects via WhatsApp groups and other applications. The questionnaire was tested in a pilot study for validity, edited, and validated by 2 supervisors at the College of Dentistry, Qassim University, Buraydah, Saudi Arabia. The questionnaire consisted of questions regarding sociodemographic attributes, the level of expertise of dental practitioners, and their knowledge and perspectives regarding COVID-19 and the implementation of oral lesions interrelated to it. The data was subjected to analysis through the utilization of descriptive statistics and chi-square tests, employing the statistical software SPSS (version 24).

**Results:**

About 43.9% of subjects stated that COVID-19 causes oral symptoms. Respondents most frequently reported COVID-19-related dry mouth. Oral ulcers, Candida infection, Hyperpigmentation, tongue coating, atrophy, Petechiae, Herpes, white lesions, Gingivitis, and Periodontitis are further symptoms. COVID-19’s oral manifestation was unknown to 41.0% of subjects, and 37.7% of respondents lacked knowledge regarding the most impacted locations of oral manifestations. Oral signs and COVID-19 symptoms are debated and significantly associated with higher educational levels.

**Conclusion:**

The dental students and freshly graduated dentists in this study have proper knowledge of COVID-19 and its symptoms. Also, most dental students and newly graduated dentists recognize the potential correlation between COVID-19 and oral manifestations with an average to excellent knowledge of the types and sites commonly affected. The level of awareness was associated with higher educational levels.

**Supplementary Information:**

The online version contains supplementary material available at 10.1186/s12903-023-03666-9.

## Background

The newly discovered coronavirus illness (COVID-19) has recently become a worldwide pandemic Human-to-human transmission burden. According to the World Health Organization (WHO), there have been over 697 million confirmed cases of COVID-19 and over 6.9 million deaths reported worldwide since 2019 [1]. In addition to a high body temperature and dry cough, some people may also have dyspnea, impaired smell, and difficulty tasting [2].

Patients with COVID-19 have conveyed several oral lesions. From plaques on the tongue to inflammation of the papillae of Wharton’s duct, the spectrum of symptoms is broad [3–5].

The exact pathogenesis of oral symptoms in COVID-19 patients has yet to be fully understood. However, some studies suggest that the angiotensin-converting enzyme 2 (ACE2) cell receptors, which are abundantly regulated on the oral mucosa, play a role in allowing the severe acute respiratory syndrome-coronavirus-2 (SARS-CoV-2) to infect them [6]. According to an additional investigation, the potential pathogenic elements of SARS-CoV-2 are identified as its four structural proteins, with particular emphasis on the S protein. This protein exhibits a binding affinity towards human ACE2 receptors, thus permitting the virus’s entry to the host cells [4].

Recent academic papers have reported on the oral manifestations of COVID-19, including taste dysfunction, xerostomia, gingival inflammation, dysgeusia, erythema, petechiae, candidiasis, and ulcerative oral lesions of the mucous membrane [3, 7, 8]. Another systematic review with meta-analysis found that the categorization of oral lesions in COVID-19 was based on their underlying causes, encompassing iatrogenic lesions resulting from intubation procedures and accidental infections [9].

A case report emphasized the significance of incorporating dentists into the multi-professional team within the intensive care unit. This inclusion is crucial for enhancing oral health among critically ill patients, encompassing those affected by COVID-19 and aiding evidence-based decision-making in managing infectious diseases [8].

Dentists can help to identify and classify the oral ulcerative lesions in COVID-19 patients, which can help to understand the possible pathogenesis and differential diagnosis of these lesions and monitor their evolution and response to treatment; thus, a better level of knowledge and understanding of the lesions related to COVID-19 is mandatory [10].

Also, dentists’ awareness can help to prevent and control COVID-19 infection and transmission in the oral health care setting by following the recommended preventive measures and precautions [11, 12]. These are some possible reasons why it is important to include dentists in studying and treating oral lesions among covid 19 patients. However, scarce studies address the importance of dentists’ awareness regarding oral manifestations related to pandemics such as COVID-19; it is essential to study their knowledge regarding this subject. This study measures undergraduate dental students, interns, and freshly graduated dentists’ understanding of oral manifestations associated with COVID-19 in Saudi Arabia.

## Methods

### Study design and setting

A cross-sectional descriptive study was directed in Saudi Arabia from January 2022 to December 2022.

### Sample size and population

The research encompassed a cohort of undergraduate dental students (4th and 5th dental students) and freshly graduated dentists residing in the Kingdom of Saudi Arabia. The inclusion criteria were undergraduate dental students, interns, and freshly graduated dentists only from Saudi Arabia (KSA). The exclusion criteria included the dental students at 1st – 3rd year, non-Saudi citizens, uncomplete questionnaire, and participants of the pilot study. The determination of sample size depended on the number of responses using a multistage cluster sampling technique with a 95% confidence level and a 5% margin of error involving assuming a population percentage that yields the most practical sample size (*P* = 0.50). To address the potential effects of clustered approach, non-response, and insufficient information, an additional 10% of the respondents were incorporated into the study. The study’s sample size comprises 305 individuals.

### Ethical consideration

The IBR approval NO. (EA/6142/2021) of the Dental Ethics Committee, Dental Research Center, College of Dentistry, Qassim University. All methods were carried out in accordance with declaration of Helsinki, and Al-Qassim Dentistry College guidelines and regulations, and all authors declare that an online informed consent was obtained from the participants prior to conducting this research. The questionnaire was approved and validated by two supervisors of Al-Qassim Dentistry College and tested in a pilot study to ensure its clarity and comprehension [13].

### Data collection methods, instruments used, and measurements

The questionnaire inquiries were formulated subsequent to an extensive review of relevant literature and the global standards [5, 6, 14]. The survey instrument was developed in the English language. The concepts of validity and reliability are crucial in the field of research and measurement. The survey instrument was created and comprised of three distinct sections with a set of inquiries that focused on sociodemographic attributes, the level of expertise of dental practitioners, and their knowledge and perspectives regarding COVID-19 and the implementation of oral lesions related to it. The survey utilized an organized multiple-choice survey that was categorized into distinct sections. These sections included the awareness of COVID-19’s seriousness, symptoms, medications, oral lesions related to COVID-19 or its drugs, types of oral lesions, and their management. A panel of respected professionals at the College of Dentistry, Qassim University, thoroughly evaluated the questionnaire. This approach validated the content validity of the questionnaire. The Cronbach’s alpha coefficient for the revised questionnaire was satisfactory (0.7) [15].

Also, the questionnaire underwent testing by being sent to a sample of dental students and interns who were selected from the same population. These individuals had already completed the same questionnaire one month before the research. The purpose of this testing was to assess the suitability, comprehensibility, and coherence of the questions. The feedback acquired from the first survey was used to enhance the questionnaire and streamline items that were seen as unclear.

The questionnaire was created using Google Forms and disseminated to approximately 500 subjects via WhatsApp groups and other applications. The questionnaire was initiated on March 17th and terminated on April 3rd, 2022. Participation was discretionary, and the data furnished by the respondents was handled with confidentiality. The questionnaire’s respondents were afforded the liberty to discontinue their participation in the survey questionnaire at any juncture. The complete and correct answers take two marks, the right but not complete answer takes one mark, and the wrong answer takes zero.

### Data management and analysis plan

The information was gathered and inputted then the statistical assessment was conducted utilizing the Statistical Package of Social Sciences (SPSS/version 24) software. The statistical analysis method is as follows: The Chi-square (X^2^) test is utilized to compare the distribution of subjects based on various study items. Descriptive statistics, including means and standard deviations, were employed to characterize constant variables, while percentages were utilized to depict the categorical information.

## Results

### Characteristics of included subjects

Most participants are 5th-year dental students, accounting for 34.8%. Dental Interns come in second with 28.5%, followed by 4th-year dental students with 20.0% and freshly graduated dentists with 16.7% (Table 1).

**Table 1:** Distribution of studied group regarding gender and Academic year

Gender	Number	Percent
Female	126	41.3
Male	179	58.7
Total	305	100.0
**Academic year**	**Number**	**Percent**
4th -year dental students	61	20.0
5th -year dental students	106	34.8
Freshly graduated dentists	51	16.7
Dental Interns	87	28.5
**Total**	**305**	**100.0**

### Assessment of included subjects’ knowledge

Table 2 provides the distribution of the study participants’ answers to the questions related to their COVID-19 and oral lesions awareness questionnaire. The majority of the participants either strongly agree or agree that COVID-19 is a hazardous disease, accounting for a combined total of 85.2%. As for the symptoms of COVID-19 and the percentage of respondents’ knowledge of each symptom, the most common chosen symptoms are loss of taste and smell (90.8%), fever (84.9%), and dry cough (75.7%). Other symptoms include wet cough (18.0%), pain all over the body (39.3%), difficulty in breathing (32.8%), wet cough, Sneezing, sore throat (47.2%), and sneezing (35.1%). However, all the answers were correct except for wet cough and sneezing, only 21.3% of the participants have a proper and complete understanding of the symptoms, while 34.4% have a wrong understanding. It is important to note that 33.1% of subjects did not know which medication for COVID-19 (Table 3). The data indicated that a small percentage of participants, 12.8%, have a right and complete understanding of the medication prescribed. Additionally, a notable proportion of participants, 26.2%, are uncertain about their understanding of the medication prescribed to COVID-19 patients. Also, it is important to note that COVID-19’s oral manifestation was unknown to 41.0% of subjects, and 37.7% of respondents lacked knowledge regarding the most impacted locations of oral manifestations.

**Table 2:** Distribution of the studied group knowledge regarding different items of awareness

	Number	Percent
**COVID-19 is a highly dangerous disease**		
Strongly agree	116	38.0
Agree	144	47.2
Disagree	41	13.4
I don’t know	4	1.3
**The patient is suspected to be COVID-19 positive if he is suffering from** ^(*)^		
Fever	259	84.9
Dry cough	231	75.7
Wet cough	55	18.0
Loss of taste and smell	277	90.8
Pain all over the body	120	39.3
Difficulty in breathing	100	32.8
Sore throat	144	47.2
Sneezing	107	35.1
I don’t know	0	0.0
**Is there a relationship between COVID-19 and oral manifestations?**		
Yes	134	43.9
No	24	7.9
I don’t know	147	48.2
**Medications prescribed to patients COVID-19** ^(**)^		
Zithrocin	14	4.6
Iverzine	11	3.6
Zinc, vitamin C	105	34.4
Prednisolone	15	4.9
Remdesiv ir	17	5.6
Anticoagulant	8	2.6
Antihypertensive	0	0.0
Antibacterial	17	5.6
Foradil	6	2.0
Colchicine or hydroxychloroquine	19	6.2
Acetylcysteine	6	2.0
Silymarin	4	1.3
oral ulcers	98	32.1
I don’t know	101	33.1
**Is there a relationship between oral manifestations and medications taken by COVID-19 patients?**		
Yes	87	28.5
No	36	11.8
I don’t know	182	59.7
**What do you think about oral manifestations associated with COVID-19 patients?** ^(***)^		
oral ulcers	98	32.1
Candida infection	62	20.3
Hyperpigmentation	7	2.3
tongue coating	57	18.7
atrophy of the tongue	7	2.3
Petechiae	13	4.3
Herpes	23	7.5
white lesion	23	7.5
dry mouth	151	49.5
Gingivitis	61	20.0
Periodontitis	41	13.4
I don’t know	125	41.0
**The sites that you can observe the oral manifestations commonly?** ^(****)^		
Dorsal surface of tongue	115	37.7
Ventral surface of tongue	38	12.5
Lips	110	36.1
Vestibule	63	20.7
Floor of the mouth	35	11.5
Palate	67	22.0
Uvula	27	8.9
I don’t know	115	37.7
**According to manifestations do you think is it?**		
Symptomatic	51	16.7
A Symptomatic	77	25.2
Both	100	32.8
I don’t know	77	25.2
**When do you think the oral manifestations appear?**		
With the symptoms of COVID-19	99	32.5
After the symptoms of COVID-19	101	33.1
Before the symptoms of COVID-19	31	10.2
I don’t know	74	24.3
**How do you think it subside?**		
Professional intervention	43	14.1
Self-limiting	213	69.8
I don’t know	49	16.1

(*)The right and complete answer was all the right symptoms which include (fever, dry cough, Loss of taste and smell, Pain all over the body, Difficulty in breathing and sore throat) The right but not complete answer, less than 6 correct answer. The wrong answer was wet cough, Sneezing,

(**) The right and complete answer *is all the right medication which include all the medication above except antihypertensive, antibacterial, silymarin or I don’t know*

(***) The right and complete answer *was all the right oral manifestation which include all the manifestation above except Candida infection, Hyperpigmentation, tongue coating atrophy of the tongue, Petechiae or I’ don’t know*

(****)The right and complete answer was all the right observe oral manifestation which include all the upper side. The right but not complete answer, less than all correct answer. The wrong answer was I’ don’t know

**Table 3:** Distribution of studied group regarding the level of awareness

Level of awareness	Number	Percent
Excellent	61	20.0
Average and above average	103	33.8
Below average	141	46.2
**Total**	**305**	**100.0**

According to the respondents’ knowledge of the most common sites, 37.7% of respondents did not know which sites were commonly affected and 34.4% did not know the answer.

The participants have varying opinions on whether oral manifestations are symptomatic, asymptomatic, or both. Most participants, 32.8%, believe oral manifestations can be symptomatic and asymptomatic. A significant proportion of participants (69.8%) believe that oral manifestations in COVID-19 patients will resolve without intervention.

As for the total level of awareness, the participants exhibit varying degrees of awareness. 20.0% of participants have an excellent level of awareness, 33.8% have an average or above-average level of awareness, and 46.2% have a below average level of awareness (Table  3).

### Correlation between demographics and level of knowledge

The relation of awareness levels across genders is presented in Table 4. Among participants with a below-average level of awareness, 36.2% are female and 63.8% are male. Among participants with an average or above-average level of awareness, 45.6% are female and 54.4% are male. Among participants with an excellent level of awareness, 45.9% are female and 54.1% are male. The chi-squared test shows that the difference in the distribution of awareness levels across genders is not statistically significant (X2 = 2.860, *p* = 0.239).

**Table 4:** Relation between general score of awareness and gender

Gender						
	Below average		Average and above average		Excellent	
	No	%	No	%	No	%
Female	51	36.2	47	45.6	28	45.9
Male	90	63.8	56	54.4	33	54.1
**Total**	**141**	**100.0**	**103**	**100.0**	**61**	**100.0**
X^2^ P	2.860 0.239 N.S.					

The percentage of excellent students and dentists increases as the academic year increases, from 23% in 4th year to 39.3% in dental interns. This suggests that the students and dentists achieve higher levels of excellence over time. The gap between the below average and excellent categories widens as the academic year increases, from 5.3% in 4th year to 16.6% in dental interns. This suggests that there is more variation in performance among the students and dentists in later years (Table 5).

**Table 5:** Relation between general score of awareness academic year

Academic year	Below average		Average and above average		Excellent	
	No	%	No	%	No	%
4th year dental students	25	17.7	22	21.4	14	23.0
5th year dental students	48	34.0	38	36.9	20	32.8
Freshly graduated dentists	36	25.5	12	11.7	3	4.9
Dental Interns	32	22.7	31	30.1	24	39.3
**Total**	141	100.0%	103	100.0%	61	100.0%
X2 P	18.433 0.005*					

## Discussion

The oral mucosa has been identified as a significant spot of contagion for SARS-CoV-2 and is considered to be highly susceptible to COVID-19 infection. The etiology of oral manifestations remains uncertain, as it is yet to be determined whether they arise from COVID-19 infection, underlying systemic illness, or compromised immune response [16]. The impartial of the present investigation was to estimate the comprehension of dental school students, both at the undergraduate and freshly-graduate levels, regarding the oral manifestations that are exhibited by individuals afflicted with COVID-19.

### Principal findings compared to literature

The present study focused on assessing the knowledge of dentists regarding COVID-19 related oral lesions. Overall, the dentists demonstrated a satisfactory level of knowledge, which is consistent with prior research about MERS-CoV [17–19]. The findings indicate that certain deficiencies exist in understanding the SARSCoV-2 virus with respect to accurate symptomatology and pharmacological interventions. However, research found that dentists highlighted a deficiency in readiness to address a highly contagious respiratory illness like COVID-19. The study posits a need for enhanced protective measures in the dental operatory and efficient operational management and guidelines [20].

In terms of the oral lesion’s symptoms, sites, types, timing and prognosis, the level of knowledge was average to excellent among more than half of the respondents. Although the study identified a gap in dentists’ understanding of oral lesions specifically related to COVID-19, limiting comparisons to the existing literature. Nevertheless, compared to other studies on the level of knowledge about oral lesions, this study found that the awareness level among dentists was better [21–23]. According to literature, the symptoms of COVID-19-associated oral lesions can vary depending on the type of lesion, can occur on any part of the oral mucosa and can occur at any time during the course of the disease. However, they are most common in the early stages of infection [24, 25]. The prognosis of COVID-19 associated oral lesions is generally favorable, with most lesions resolving spontaneously within a few weeks without requiring specific treatment. However, the duration of these lesions can vary depending on the type of lesion and the severity of the underlying COVID-19 infection [26].

The management of COVID-19 associated oral lesions primarily focuses on symptom relief and maintaining oral hygiene. For more severe lesions, systemic medications such as corticosteroids or immunosuppressants may be considered [27]. Estimates of the prevalence of COVID-19 associated oral lesions vary widely due to differences in study methodologies and patient populations. However, studies suggest that oral lesions may affect a significant proportion of COVID-19 patients, with some reports indicating a prevalence of up to 30% [24, 28].

However, due to the non-specific nature of oral lesions, it is essential to consider other potential causes in the differential diagnosis. These include herpes simplex virus (HSV), aphthous stomatitis, hand, foot, and mouth disease (HFMD), candidiasis, lichen planus, trauma, allergic reactions, nutritional deficiencies, and other medical conditions. A thorough medical history, physical examination, and appropriate laboratory tests are crucial for establishing an accurate diagnosis and guiding appropriate management [24, 26, 29].

A study conducted in Istanbul, Turkey in 2009 revealed that a significant proportion of dentists faced challenges in accurately diagnosing oral mucosal lesions, with approximately 85% experiencing difficulties in this regard. Furthermore, approximately 62% of the dentists surveyed did not actively seek to update their knowledge through literature sources. Additionally, a substantial majority of dentists, approximately 93%, did not engage in the practice of conducting biopsies or seeking consultation from other healthcare professionals when faced with such lesions. Thus, most dentists had trouble diagnosing oral mucosal lesions [22].

On a positive note, recent research conducted at Princess Nourah University in Riyadh, Saudi Arabia, revealed that healthcare providers there were informed about oral squamous cell carcinoma, a type of oral cancer. The practitioners demonstrated an understanding of risk indicators for the disease. However, the study also highlighted that a lack of training was the main obstacle preventing a comprehensive oral examination for early identification of oral squamous cell carcinoma. This suggests that while there is some awareness and knowledge of this type of oral cancer among healthcare providers, there is still room for improvement in terms of training and conducting thorough examinations. It is essential for healthcare providers to receive adequate training and education to ensure early identification and appropriate management of such conditions to provide optimal patient care [23].

Our research encountered no disparity between genders in oral mucosal lesion knowledge, but freshly graduated dentists answered more questions correctly than students which is consistent with another study conducted in KSA to evaluate the general knowledge of students and interns regarding oral lesions’ knowledge [21]. A study at Ajman University found that dental interns (52.5%), final year students (44.1%) and general dentists (51.9%) had the highest rate of correct answers. There were no gender differences in their ability to classify and distinguish correct answers. These results may be attributed to the fact that freshly graduates care for patients more comprehensively and their dedicated efforts in studying for licensure examinations. These results reinforce continuous education [30].

Al-Kharj researchers found that males had somewhat more knowledge than females, although the difference was minor. Contrary to our results, they observed no significant influence of graduation time on knowledge. Most dentists were ignorant of frequent etiologic variables, high-risk areas, and what to assess during a normal visit [31]. This research demonstrated that clinicians might miss clinical symptoms for all oral lesions.

About 43.9% of subjects stated that COVID-19 causes oral symptoms. The logical link between oral lesions and COVID-19 has been testified in the literature [32–35]. The inflammation triggered by the virus can induce tissue damage directly and indirectly, facilitating the emergence of other complications, such as heightened hypersensitivity. Furthermore, the identification of ACE2 receptors in the oral mucosa [36] and the presence of viral particles in saliva [37] may provide evidence or at least suggest the plausibility of authentic oral manifestations of COVID-19 [19, 20]. Several studies have documented the incidence of oral lesions at an early stage of the disease [33, 34] or their manifestation in the absence of typical systemic symptoms [32].

The most commonly reported oral symptom related to COVID-19 was dry mouth. Other symptoms included oral ulcers, Candida infections, hyperpigmentation, tongue coating, atrophy, petechiae, herpes, white lesions, gingivitis, and periodontitis [32, 38]. Interestingly, a significant proportion of patients (41.0%) were unaware of COVID-19’s oral manifestations, and a similar percentage (37.7%) did not know which locations in the mouth were most affected.

The World Health Organization (WHO) has recently included gustatory impairment, in addition to olfactory changes, as a recognized symptom of COVID-19. Patients diagnosed with COVID-19 may exhibit various types of oral lesions, including but not limited to ulcerative, erosive, vesicobullous, and plaque-like presentations [6, 39].

Oral mucosal inflammation may manifest concomitantly with typical manifestations of COVID-19 or supplementary skin presentations. The appearance of lesions is observed concomitantly with or preceding systemic manifestations of COVID-19 [6]. The correlation amongst COVID-19 and oral lesions remains uncertain and requires further investigation. Several publications have reported that taste impairment is the sole oral symptom associated with COVID-19. In contrast, other oral lesions may arise due to factors such as decreased immunity resulting from a viral infection, opportunistic or recurrent infection, or medical management for COVID-19 [29, 40]. It has also been observed that these oral mucosal lesions tend to exhibit disappearing symptoms or a reduction in size over time, usually within 6 to 14 days It is still uncertain whether there is a direct correlation between these oral lesions and COVID-19, or if they may be caused by other factors such as decreased immunity or medical management for the disease [8, 41].

### Strengths and limitations

Strengths: This study is one of the few that have explored the dentists’ knowledge of oral lesions associated with COVID-19 pandemic and emphasized the importance of their role in oral health during such crises. The sample size was adequate and the questionnaire was validated which would help researchers for future research.

This study was subject to a number of limitations. The introduction of a novel questionnaire was accompanied by a predominantly undergraduate and recent graduate participant pool, necessitating consideration of this demographic when analyzing the results. It is recommended that a subsequent survey be conducted, which should comprise of more comprehensive questionnaires and a larger sample size of institutions and participants, with a particular focus on recently graduated dentists, to authenticate the findings of the current study. Also, this study does not include data on the subjects’ practice or knowledge regarding management options, and medications’ doses.

### Implications and future studies

Additional investigation is required to validate a potential association between documented mucosal lesions and COVID-19, as these lesions may serve as an initial indication of the disease or may be a result of various factors such as medication usage, compromised immunity, localized or widespread inflammation, vascular impairment, and inadequate oral hygiene. It is imperative for dental practitioners to possess knowledge regarding oral symptoms, influencing factors, and fundamental mechanisms prior to conducting patient examinations and commencing treatment.

Further studies are required to comprehend better the prevalence and causal relationships between oral lesions and COVID-19. Also, it is important to recognize the pathogenesis of oral manifestations in COVID-19 patients and increase dentists’ knowledge to enhance their ability to pandemic challenges associated with oral lesions.

## Conclusion

The dental students and freshly graduated dentists in this study have proper awareness of COVID-19 and its symptoms. Also, most dental students and freshly graduated dentists recognize the potential correlation between COVID-19 and oral manifestations with an average to excellent knowledge of their types and sites commonly affected. The level of awareness was associated with higher educational levels.

### Electronic supplementary material

Below is the link to the electronic supplementary material.

Supplementary Material 1

## Data Availability

The data are available from the corresponding author upon request.
