# Supplementary material for: Awareness of undergraduate dental students, interns, and freshly graduated dentists about oral manifestation associated with COVID-19
Source: BMC Oral Health. 2023 Dec 9;23:990. doi: 10.1186/s12903-023-03666-9 (PMC10710720; doi:10.1186/s12903-023-03666-9)
Supplement: Supplementary file 1 — Supplementary Material 1 [file 12903_2023_3666_MOESM1_ESM.doc]

**Students' Research and Presentation Skills Supervision Committee**

**SRPSSC**

**Original Research Proposal Form**

1. Gender

- Male
- Female

2. Academic year

- 4th years dental student
- 5th years dental student
- Dental Interns
- Freshly graduated dentists

3. Institution:

4. COVID-19 is a highly dangerous disease

- Strongly agree
- Agree
- I don’t know
- Disagree
- Strongly disagree

5. Patient is suspected to be COVID-19 positive if he is suffering from

- Fever
- Dry cough
- Wet cough
- Loss of taste and smell
- Pain all over the body Difficulty in breathing
- Sore throat
- Sneezing
- I don’t know

6. Is there a relationship between COVID-19 and oral manifestations?

- Yes
- No
- I don’t know

7. Medications prescribed to the patients COVID-19?

- Zithrocin
- Iverzine
- Zinc, vitamin C
- Prednisolone
- Remdesiv ir
- Anticoagulant
- Antihypertensive
- Antibacterial
- Foradil
- Colchicine or hydroxychloroquine
- Acetylcysteine
- Silymarin
- I don’t know

8. Is there a relationship between oral manifestations and medications taken by COVID-19 patients?

- Yes
- No
- I don’t know

9. what do you think about oral manifestations associated with COVID-19 patients?

- oral ulcers
- Candida infection
- hyperpigmentation
- tongue coating
- atrophy of the tongue
- petechiae
- herpes
- white lesion
- dry mouth
- gingivitis
- periodontitis
- I don’t know

10. the sites that you can observe the oral manifestations commonly?

- Dorsal surface of tongue
- Ventral surface of tongue
- Lips
- Vestibule
- Floor of the mouth
- Palate
- Uvula
- I don’t know

11. According to manifestations do you think is it?

- Symptomatic
- A Symptomatic
- Both
- I don’t know

12. When do you think the oral manifestations appear?

- Before the symptoms of COVID-19
- With the symptoms of COVID-19
- After the symptoms of COVID-19
- I don’t know

13. How do you think it subside ?

- Self limiting
- Professional intervention
- I don’t know
